# TargIDe: a machine-learning workflow for target identification of molecules with antibiofilm activity against *Pseudomonas aeruginosa*

**DOI:** 10.1007/s10822-023-00505-5

**Published:** 2023-04-22

**Authors:** João Carneiro, Rita P. Magalhães, Victor M. de la Oliva Roque, Manuel Simões, Diogo Pratas, Sérgio F. Sousa

**Affiliations:** 1 Interdisciplinary Centre of Marine and Environmental Research, CIIMAR, University of Porto, Terminal de Cruzeiros do Porto de Leixões, Av. General Norton de Matos, s/n, Porto, 4450-208 Portugal; 2 Faculty of Medicine, Associate Laboratory i4HB—Institute for Health and Bioeconomy, University of Porto, 4200-319 Porto, Portugal; 3 Department of Biomedicine, Faculty of Medicine, UCIBIO—Applied Molecular Biosciences Unit, University of Porto, BioSIM, Porto, 4200-319 Portugal; 4 Faculty of Engineering, LEPABE Laboratory for Process Engineering, Environment, Biotechnology and Energy, University of Porto, Rua Dr. Roberto Frias, s/n, Porto, 4200-465 Portugal; 5 Faculty of Engineering, ALiCE—Associate Laboratory in Chemical Engineering, University of Porto, Rua Dr. Roberto Frias, 4200-465 Porto, Portugal; 6 Institute of Electronics and Informatics Engineering of Aveiro, IEETA, University of Aveiro, Aveiro, Portugal; 7 Department of Electronics, Telecommunications and Informatics, DETI, University of Aveiro, Aveiro, Portugal; 8 Department of Virology, DoV, University of Helsinki, Helsinki, Finland

**Keywords:** Biofilms, *Pseudomonas aeruginosa*, Machine learning, Ligand targets

## Abstract

Bacterial biofilms are a source of infectious human diseases and are heavily linked to antibiotic resistance. *Pseudomonas aeruginosa* is a multidrug-resistant bacterium widely present and implicated in several hospital-acquired infections. Over the last years, the development of new drugs able to inhibit *Pseudomonas aeruginosa* by interfering with its ability to form biofilms has become a promising strategy in drug discovery. Identifying molecules able to interfere with biofilm formation is difficult, but further developing these molecules by rationally improving their activity is particularly challenging, as it requires knowledge of the specific protein target that is inhibited. This work describes the development of a machine learning multitechnique consensus workflow to predict the protein targets of molecules with confirmed inhibitory activity against biofilm formation by *Pseudomonas aeruginosa*. It uses a specialized database containing all the known targets implicated in biofilm formation by *Pseudomonas aeruginosa.* The experimentally confirmed inhibitors available on ChEMBL, together with chemical descriptors, were used as the input features for a combination of nine different classification models, yielding a consensus method to predict the most likely target of a ligand. The implemented algorithm is freely available at https://github.com/BioSIM-Research-Group/TargIDe under licence GNU General Public Licence (GPL) version 3 and can easily be improved as more data become available.

**Supplementary Information:**

The online version contains supplementary material available at 10.1007/s10822-023-00505-5.

## Introduction

Microbial biofilms are complex consortia of bacteria embedded in a self-produced extracellular matrix. These microbial biofilms can adhere to biological or nonbiological surfaces and differ greatly from their planktonic (single cells primarily in suspension) counterparts since they display metabolic heterogeneity and altered gene expression [1]. Biofilms typically concentrate at an interface (most commonly solid‒liquid), and their association with surfaces is mostly irreversible, complicating their removal by mechanical force or through rinsing [2, 3]. Their formation, development and pathogenicity depend on several complex factors and mechanisms. Extracellular polymeric substances (EPS) compose the matrix that encases the cells and influences the formation and development of the biofilm.

The study of bacteria in biofilms is important for public health, considering that they are more resistant to antibiotics and the host immune response than when in the planktonic state [1]. Due to the threat that they represent to individuals, medical [4] and industrial systems [5], understanding these factors and mechanisms is critical to develop innovative multifactorial treatments against them. These threats manifest particularly in healthcare. The relationship between biofilm formation and indwelling infectious diseases is well reported in the literature. In 2017, the National Institutes of Health (NIH) estimated that over 80% of allbacteria-related infections in humans are caused by biofilms [5]. In the United States, estimates point to over 17 million new biofilm-caused infections every year, resulting in 550,000 annual deaths [4, 5]. Most tissue- and device-related biofilm infections acquired in a hospital setting are caused by a relatively short list of bacteria: *Staphylococcus aureus* [6], *Pseudomonas aeruginosa (P. aeruginosa)* [7], *Escherichia coli* [8], *and Klebsiella pneumoniae* [9], among others [10].

As the prevalence of biofilms in clinical settings increases, so does the urgency to develop specific therapeutic strategies against these bacteria. Their increased antimicrobial resistance imposes a challenge in the development of drug therapies against these structures [11]. *P. aeruginosa* is an aerobic rod-shaped gram-negative opportunistic pathogen. It forms biofilms and is responsible for a wide range of diseases in humans, in addition to causing up to 20% o hospital infections. *P. aeruginosa* in biofilms display higher resistance to external therapies and host defences, making their treatment extremely difficult [7, 12]. The majority of *P. aeruginosa* infections are observed in patients with cystic fibrosis (CF) and chronic obstructive pulmonary disease (COPD) [13]. However, they are also involved in several urinary tract and nosocomial infections, in addition to invading medical devices such as prosthetic joints and catheters. Most of these infections have serious health-related consequences [7, 12]. *P. aeruginosa* pathogenicity is highly complex and depends on several virulence factors, and the molecular components related to biofilm formation and cellular attachment and adhesion present in *P. aeruginosa* can be considered possible targets to combat its pathogenicity [7, 12].

As such, in recent years, several studies have focused on the identification and development of molecules able to inhibit biofilm formation and development in *P. aeruginosa* [14–20]. While different experimental approaches can be used to this aim, identifying the particular protein target on which the given molecule is acting is significantly more difficult from a technical and economic perspective. Correctly identifying the protein target associated with the inhibitory activity of such molecules is essential for rational optimization of their activity. Hence, the development of methods able to pair molecules with confirmed *P. aeruginosa* antibiofilm activity and their putative protein targets is of the utmost importance.

In our previous works [21, 22], we dedicated particular attention to seven key protein targets associated with biofilm formation and resistance in *P. aeruginosa*, namely, LasR, PqsA, PqsD, PqsR, RhIR, ExsA and LecB. LecB is involved in the adherence to target host cells [23], while the rest are involved in quorum-sensing (QS)/cell-to-cell communication. Using QS, bacteria can alter gene expression depending on the population density, and as such, QS pathways and biofilm formation are tightly related [24]. In *P. aeruginosa*, there are four main QS systems. LasR and RhIR are transcriptional receptors from two different systems (the *las* and *rhr* systems, respectively), which bind autoinducers produced by each system synthase, inducing biofilm formation. PqsD, PqsA and PqsR all belong to the *pqs* system and are part of a QS activating cascade. The *las* system is the main regulator since it activates both the *rhr* and *pqs* systems [12, 25]. ExsA is a transcriptional activator involved in the *P. aeruginosa* type III secretion system and is actively involved in biofilm formation [26].

Machine learning (ML) methods have been acquiring a growing importance in drug discovery efforts [27–30]. The applications of ML in drug discovery are multivariate, both integrated with complementary computer-aided drug discovery techniques such as molecular docking [31–33] and virtual screening [34–36] or being used on their own [37–40]. Furthermore, the growing availability of biological and molecular data in online databases [41] allows the development of more specific and successful models. ML models can be divided into supervised learning (SL), where the training data include both the input and the target or desired results, unsupervised learning (UL), mostly used for clustering purposes, and semisupervised models, which use labelled and unlabelled samples to improve the performance of the model [42–44]. SL can be used for classification, regression and clustering purposes, and several different models and techniques can achieve the same purpose [45].

Popular ML models include the K-nearest neighbours (KNN), support vector machines (SVM), neural networks (NN), naïve Bayes classifier (NBC), random forest (RF), and XGBoost. KNN is a model that compares the features of the unknown molecule with those of the closest k-neighbour molecules and predicts a similar target. It is based on the assumption that molecules with similar features will have similar target-binding behaviours [46]. In SVM, a separating hyperplane based on the input features is defined, and the molecules are classified according to the side of the hyperplane they fall into. The training dataset results in the definition of the hyperplane and the thresholds of classification. The submission of an unknown molecule and respective features leads the model to place it in one of the sides, corresponding to a predicted target [46]. NNs are based on the human neuron-based nervous system, in which several neuron layers separate the input data from the output response. The constant feedback between the input layer and the hidden neurons results in learning and training, which becomes more specific as the information moves through the model, allowing for accurate predictions emitted by the output layer [47]. Naïve Bayes is another classifier that can be used to determine target-ligand pairs, as already shown by Yao et al. [48]. NBC counts the frequency of categories to predict probabilities for the features [49]. This type of classifier allows good prediction performance using a small amount of training data and can process large amounts of data with quick training times and a tolerance for noise [50–52]. RF combines different classification methods by using high-dimensional data and merging and obtaining outcomes over individual decision trees. RF procedures were previously tested efficiently on large datasets with large numbers of input variables. They are relatively insensitive to noise and outliers [49, 53]. RF methods have also been applied to understand relationships between drugs over cell lines recurring to genomic information, drug targets and pharmacological information [53]. XG-Boost is an efficient and scalable variant of the gradient boosting machine [54] that can be easily parallelized and has shown a high predictive accuracy. XG-Boost is characterized by an intrinsic ability to handle complex descriptor feature spaces, especially in cases where there is an imbalance in class distribution [55]. XG-Boost was used by Xing et al. [56] to detect molecules for targets involved in rheumatoid arthritis, and Mustapha et al. [57] implemented an XG-Boost model to ascertain the bioactive chemical potential of several molecules.

Here, we describe the development of an ensemble machine learning classification model to predict the most likely protein target of molecules with confirmed experimental antibiofilm activity against *P. aeruginosa*. This consensus model is based on a selection of nine ML models following initial tests involving the development of 27 different ML models using KNN, SVM, NN, NBC, RF and XGBoost.

## Methodology

### Initial database

**Fig. 1:**
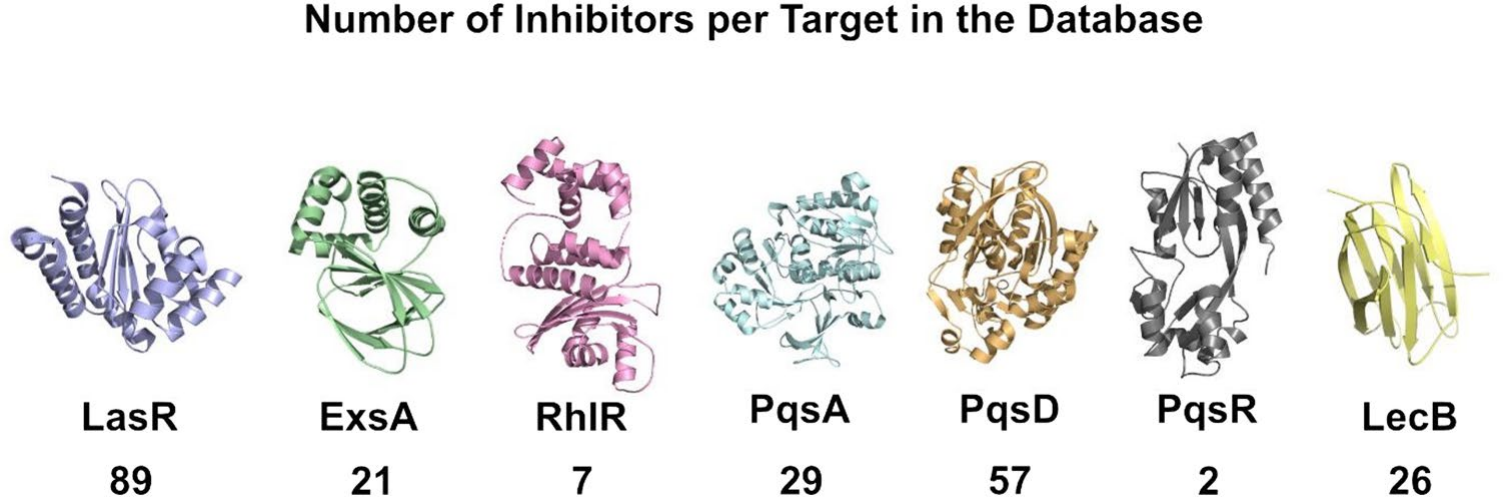
Molecular representation of the protein targets included in this study and the number of ligands used per target

The KEGG [58] database was searched for protein targets involved in biofilm formation and resistance in *P. aeruginosa.* Each of these targets was then searched in ChEMBL [59], enabling the identification of ligand-target specific activity relationships. All ligands under the “IC50” and “Inhibition” categories were downloaded, and the database was curated to include only those with relevant values. Thus, we considered ligands with an IC50 above 0 and below 600,000 nM and an inhibition percentage above 0. The final training database contained 231 ligands distributed over seven protein targets, as represented in Fig. 1. The 7 protein targets were used to build a multiclass (7) machine learning classification model.

A total of 629 chemical descriptors were calculated for each ligand using PaDEL [60] and DataWarrior software [61, 62]. These were used as input features in the classification models (Supplementary Data Table 1). Given the high and redundant number of features, a pipeline for feature reduction was applied. First, a correlation matrix between features was calculated using the coefficient of determination (Pearson’s r), and one of the features above a threshold of 0.8 was dropped. Then, recursive feature elimination with a random forest estimator and 5-fold cross-validation for feature selection (RFECV) from the scikit learn Python [63, 64] package were used to select the 10 most relevant features as obtained through RFECV. All possible combinations of 4 and 5 of these features were calculated to be used as input for model training, evaluation, and application.

### Classification models

We have used a machine learning classification-based model algorithm to predict a categorical outcome considering the targets observed in the initial dataset (TargIDe_FullDatabase_SupplementaryTable 1). The input dataset for a machine learning classification-based model typically consists of labelled examples with features and their corresponding class labels (e.g., target in our dataset). The rationale for using a machine learning classification-based model development approach is that when there is similarity between the data and the used features are sufficiently representative to classify different classes, it can accurately predict the class labels of new data based on the patterns learned from the training data. All models were developed with scikit learn Python packages. For model development and evaluation, the database was split into two sets generated with random sampling: (a) training (70%) and testing (30%); (b) training (80%) and testing (20%); (c) training (90%) and testing (10%). Several different machine learning classification methods were developed and employed, namely, KNN, NN, SVM with 4 different kernels, NBC, RF, and XGBoost. The models were trained several times, first with the 10 most relevant features, and afterwards with combinations of 4, 5, and 6 of these features using median values of information gain (IG), IG ratio, Gini decrease, and chi-square (**χ**^2^). After training, predictions for the training and test sets were calculated. The metrics used to evaluate the models were the F1 score, Jaccard score, accuracy, precision and recall. These metrics were calculated for both the training and test set predictions. To select the better performing models and combinations, an average of all the metrics was calculated, and the results with the highest average were considered. The better performing combinations of 4 and 5 features from each model were used to re-evaluate the models by their attribution to a given class of two randomly selected ligands that were not part of the original training set. We also used an independent positive control database retrieved from https://bioinfo.imtech.res.in/manojk/sigmol/uniq_QSSMs.php (SigMol). SigMol is a database of Quorum Sensing Signalling Molecules that are present in prokaryotes. We cross validated this database using recipient genes as targets.

### Cross-validation ROC curves

We used the JASP [65] and Orange data mining software (https://orangedatamining.com/) metrics generated from internal 5-fold and 20-fold cross-validation after bootstrapping replicable sampling to evaluate and compare predictive performances. We ranked the features using combinations of 4 and 10 and evaluated the median values of IG, IG ratio, Gini decrease, and chi-square (**χ**^2^). We used ROC curves [66], a decision boundary matrix to evaluate the true and false positive ratios, and multidimensional scaling. The models were measured considering the area under the curve (AUC), classification accuracy (CA), F1 score, precision, and recall.

### Applicability domain

The applicability domain of our machine learning models was calculated to test if the models could make reliable predictions. The applicability domain was calculated using an in-house Python script that transforms the features used in each model in the two component vectors that better represent the data. In summary, PCA1 and PCA2 are new features that are linear combinations of the original features, where the coefficients of the linear combinations are given by the eigenvectors of the covariance matrix. These new features capture most of the variance in the original data while reducing its dimensionality. The statistical method to perform this analysis was the principal component analysis (PCA) bounding box [67]. Prediction for new compounds that fall outside of the applicability domain may not be reliable.

### Screening

The models that resulted in the most accurate inhibitor-target pairing were used to screen a collection of randomly selected ligands from the *P. aeruginosa* subset of aBiofilm [68], a database of antibiofilm agents. Since this database is a collection of biofilm inhibitors with no identified targets, it was used to test the usage, applicability, and consistency of the best performing ML models.

### Implementation

The models were developed in the Python language using Scikit-Learn [63, 64], a Python module that integrates several supervised and unsupervised machine learning algorithms. Code development was performed in Microsoft Visual Studio IDE. The final ML algorithm with the model implementation is available at https://github.com/BioSIM-Research-Group/TargIDe. The Python code was optimized to run in a workstation with 8 CPU cores and a minimum of 8 GB of RAM.

## Results and discussion

### Feature selection

**Fig. 2:**
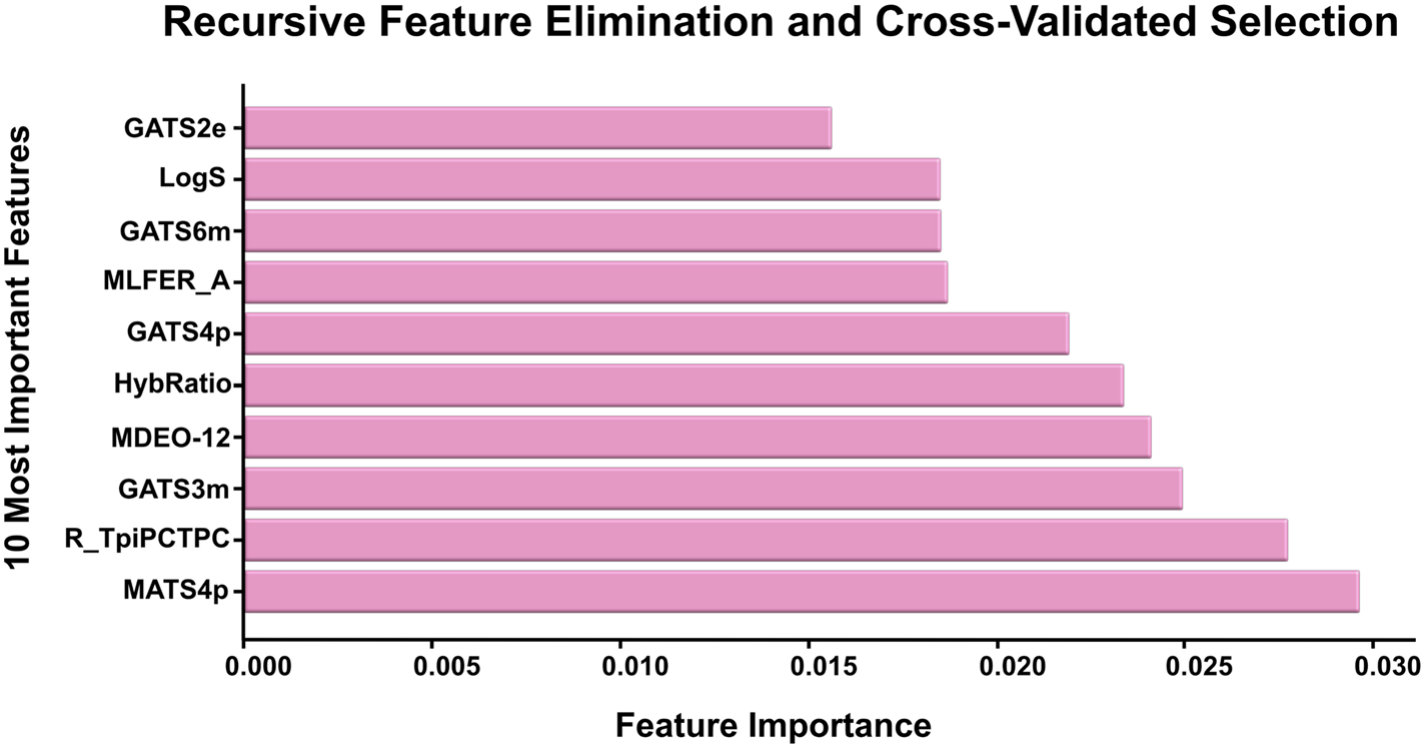
Curated training database 10 most relevant features using RF and selected after the recursive feature elimination and cross-validation process

A total of 58 protein targets involved in biofilm formation and development in *P. aeruginosa* were identified in the KEGG database. Of these, seven had associated inhibitors in ChEMBL [22, 23], namely, LasR, PqsA, PqsD, PqsR, RhIR, ExsA and LecB. These proteins are generally considered primary targets for interfering with biofilm formation and development [69]. The curated training database contained 231 ligands. The correlation matrix identified 378 features as highly correlated according to our established threshold. These were dropped from the database. The remaining 251 features were subjected to an RFECV pipeline with a random forest estimator, thereby yielding the 10 most relevant features, as represented in Fig. 2.

These features were GATS2e, GATS6m, GATS4p, GATS3m, MLFER_A, MDEO-12, LogS, MATS4p, R_tpiPCTPC and hybridization ratio. The chemical meaning of these features is described in Supplementary Data Table 1.

### Machine learning classification models evaluation

**Fig. 3:**
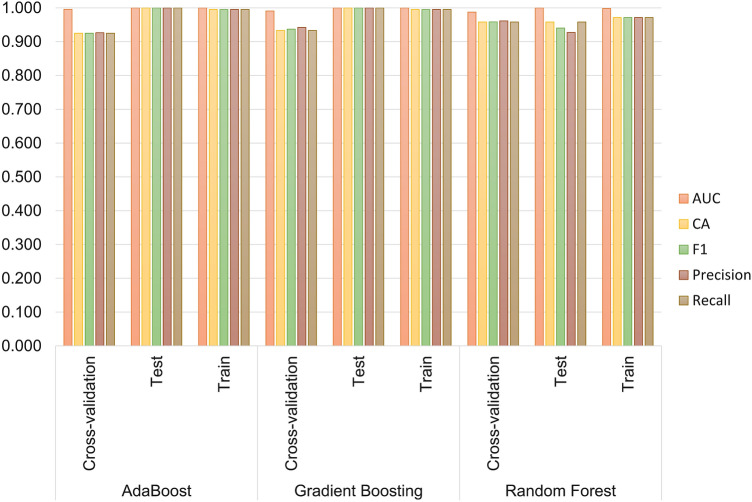
Bar graphs of the evaluation of several classification model predictions on the test (10%) and training (90%) datasets using the 10 most relevant features. The cross-validation values are also shown. Bar values for train/test/cross-validation represent, from left to right, recall, precision, F1 score, AUC. and classification accuracy (CA).

Each classification model was trained and evaluated with the 10 features mentioned and with all possible combinations of 4 and 5 of these features. All considered metrics vary between 0 and 1, with 1 indicating a better model performance, i.e., more ligands associated with the right targets [70]. Furthermore, similar predictive results for the training and test sets represent better predictions [71, 72]. In the results obtained with the 10 most relevant features for training dataset (90% of the initial dataset) and testing dataset (10% of the initial dataset) [Table 1; Fig. 3], AdaBoost, Gradient Boosting and Random Forest achieved high scores across all metrics on the train set. It is important to note that high performance on the train set does not necessarily indicate good generalization to new data. The performance on the test set is a better indicator of how well the model generalizes. The cross-validation results calculates a robust estimate of the model’s performance compared to evaluating it on a single train/test split. When comparing the train and test set the AdaBoost and Gradient Boosting achieved high scores across all metrics. KNN achieved lower scores across all metrics on the test set compared to the train set. Based on the table, it appears that AdaBoost and Gradient Boosting achieved perfect performance on the test set with an AUC of 1.00 and values of 1.00 for Recall, Precision, F1 score, and CA. KNN had lower performance on the test set with an AUC of 0.91 and values of 0.54 for Recall and CA, 0.58 for Precision, and 0.53 for F1 score. On the cross-validation set, AdaBoost had the highest performance with an AUC of 1.00 and values of 0.93 for Recall, Precision, F1 score, and CA. On the train set, both AdaBoost and Gradient Boosting achieved perfect performance with an AUC of 1.00 and values of 1.00 for Recall, Precision, F1 score, and CA. KNN had lower performance on the train set with an AUC of 0.98 and values of 0.84 for Recall and CA, 0.84 for Precision, and 0.83 for F1 score. In this case, the cross-validation results are generally consistent with the test set results. The results were very similar for the dataset splits with proportion train/test, respectively 70/30 and 80/20.

**Table 1:** Evaluation of several classification model predictions on the test and training sets using the 10 most relevant features. The bold values represent the mean values of the model considering the cross-validation, test, and train dataset

Model	Recall	Precision	F1	AUC	CA
AdaBoost	**0.97**	**0.97**	**0.97**	**1.00**	**0.97**
Cross-validation	0.93	0.93	0.93	1.00	0.93
Test	1.00	1.00	1.00	1.00	1.00
Train	1.00	1.00	1.00	1.00	1.00
Gradient boosting	**0.98**	**0.98**	**0.98**	**1.00**	**0.98**
Cross-validation	0.93	0.94	0.94	0.99	0.93
Test	1.00	1.00	1.00	1.00	1.00
Train	1.00	1.00	1.00	1.00	1.00
KNN	**0.70**	**0.72**	**0.69**	**0.94**	**0.70**
Cross-validation	0.73	0.74	0.73	0.92	0.73
Test	0.54	0.58	0.53	0.91	0.54
Train	0.84	0.84	0.83	0.98	0.84
Neural network	**0.87**	**0.85**	**0.85**	**0.98**	**0.87**
Cross-validation	0.80	0.82	0.79	0.96	0.80
Test	0.96	0.93	0.94	1.00	0.96
Train	0.84	0.82	0.82	0.97	0.84
Random forest	**0.96**	**0.95**	**0.96**	**1.00**	**0.96**
Cross-validation	0.96	0.96	0.96	0.99	0.96
Test	0.96	0.93	0.94	1.00	0.96
Train	0.97	0.97	0.97	1.00	0.97

The cross-validation results are also shown. The evaluation values are recall, precision, F1 score, AUC, and CA

**Table 2:** Table showing the 3 best evaluated models for the training and testing dataset procedures considering 4 of the 10 most relevant features. The bold values represent the mean values of the model considering the cross-validation, test, and train dataset

Model	AUC	CA	F1	Precision	Recall
Gradient boosting	**0.983**	**0.951**	**0.950**	**0.949**	**0.951**
Cross-validation	0.948	0.861	0.858	0.856	0.861
Test	1.000	0.995	0.995	0.996	0.995
Train	1.000	0.995	0.995	0.996	0.995
AdaBoost	**0.981**	**0.949**	**0.947**	**0.946**	**0.949**
Cross-validation	0.944	0.856	0.850	0.847	0.856
Test	1.000	0.995	0.995	0.995	0.995
Train	1.000	0.995	0.995	0.995	0.995
Random forest	**0.984**	**0.915**	**0.911**	**0.908**	**0.915**
Cross-validation	0.956	0.828	0.822	0.818	0.828
Test	0.999	0.962	0.958	0.958	0.962
Train	0.999	0.957	0.953	0.949	0.957

The area under the curve (AUC), classification accuracy (CA), F1 score, precision, and recall values were used to determine the models with the best accuracy

**Table 3:** Confusion matrix for XGBoost (showing proportion of actual) after cross validation (20-fold) of classification model predictions using combinations of 4 of the 10 most relevant features. The values in bold represent the sum of actual and predicted targets through the cross-validation process

	Predicted							
	ExsA	LasR	LecB	PqsA	PqsD	PqsR	RhlR	∑
Actual								
ExsA	100.00%	0.00%	0.00%	0.00%	0.00%	0.00%	0.00%	**16**
LasR	0.00%	94.10%	0.00%	2.40%	3.50%	0.00%	0.00%	**85**
LecB	0.00%	0.00%	100.00%	0.00%	0.00%	0.00%	0.00%	**27**
PqsA	0.00%	0.00%	0.00%	97.10%	2.90%	0.00%	0.00%	**35**
PqsD	1.60%	3.30%	0.00%	0.00%	95.10%	0.00%	0.00%	**61**
PqsR	0.00%	0.00%	0.00%	0.00%	0.00%	100.00%	0.00%	**2**
RhlR	0.00%	0.00%	0.00%	0.00%	0.00%	0.00%	100.00%	**5**
**∑**	**17**	**82**	**27**	**36**	**62**	**2**	**5**	**231**

**Table 4:** Evaluation metrics after the cross-validation procedure for each categorical target class present in the database considering the XGBoost machine learning classification model

Evaluation Metrics								
	ExsA	LasR	LecB	PqsA	PqsD	PqsR	RhlR	Average/Total
Support	7	33	7	8	12	1	1	69
Accuracy	0.97	0.90	1.00	0.93	0.91	0.99	0.99	0.95
Precision (Positive Predictive Value)	0.86	0.93	1.00	0.64	0.71	NaN	NaN	0.83
Recall (True Positive Rate)	0.86	0.85	1.00	0.88	0.83	0.00	0.00	0.84
False Positive Rate	0.02	0.06	0.00	0.07	0.07	0.00	0.00	0.03
False Discovery Rate	0.14	0.07	0.00	0.36	0.29	NaN	NaN	0.17
F1 Score	0.86	0.89	1.00	0.74	0.77	NaN	NaN	0.83
Matthews Correlation Coefficient	0.84	0.80	1.00	0.71	0.72	NaN	NaN	0.81
Area Under Curve (AUC)	1.00	0.86	1.00	0.92	0.94	0.00	0.68	0.77
Negative Predictive Value	0.98	0.87	1.00	0.98	0.96	0.99	0.99	0.97
True Negative Rate	0.98	0.94	1.00	0.93	0.93	1.00	1.00	0.97
False Negative Rate	0.14	0.15	0.00	0.13	0.17	1.00	1.00	0.37
False Omission Rate	0.02	0.13	0.00	0.02	0.04	0.01	0.01	0.03
Threat Score	2.00	3.11	–	0.78	1.00	0.00	0.00	–
Statistical Parity	0.10	0.43	0.10	0.16	0.20	0.00	0.00	1.00

All metrics are calculated for every class against all other classes

**Fig. 4:**
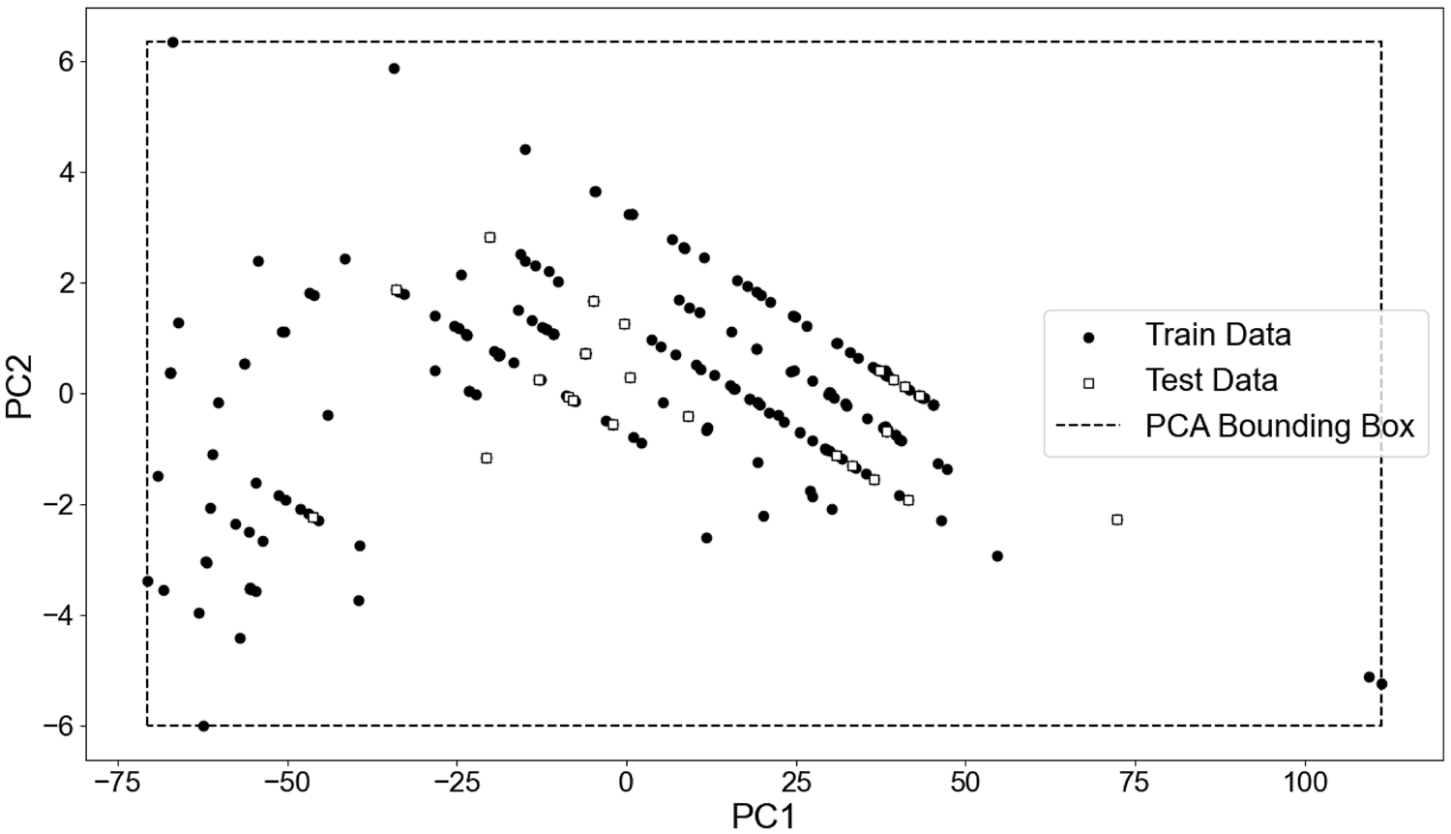
Applicability domain calculated for the train and test dataset calculated by PCA bounding box. The first principal component (PCA1) is the direction in which the data varies the most. The second principal component (PCA2) is orthogonal to the first and represents the direction of maximum variance that is not captured by the first principal component

The 3 models with the best results obtained using 4 of the most relevant chemical descriptors as input features are represented in Table 2. The results show that, similar to the previous cases, most models performed well. Interestingly, SVM with a polynomial kernel performed better than the linear model. NN resulted in the most similar predictions between the training and testing sets. AdaBoost (accuracy = 0.95), XGBoost (accuracy = 0.95), and KNN (accuracy = 0.96) were the better classifiers when using 4 input features. The results obtained with combinations of 5 and 6 features are very similar. The best performing model after the cross-validation procedure is shown in Table 3. These results show that the boosting algorithms have the highest values for the different evaluation measures. Nevertheless, the analysis through each categorical class (Table 4) for XGBoost demonstrates that a higher false positive rate (1) is observed for the targets PqsR and RhlR. All the other false positive rate values for the different classes were lower than 0.17. These results show that our methodology classifiers can be used to test thousands of compounds, circumventing the bottlenecks of laboratory experiments that involve multimillion-dollar effort [72]. We also calculated the applicability domain (Fig. 4). We showed that the test dataset do not cross the boundaries of the training dataset.

### Applicability of the machine learning models using target-molecule pairs

Considering the previously evaluated scores, we applied the models for the prediction of the target-molecule pairing for two random molecules of each of the 7 protein targets included in the model. The results for this experiment are described in Table 5.

**Table 5:** Prediction results for the target-ligand pairing between 2 randomly selected ligands for each of the 7 protein targets included in the model, using all the ML models with different numbers of features (FT).

Right target	Random ligand A							Random ligand B						
	PqsD	LasR	ExsA	RhlR	LecB	PqsR	PqsA	PqsD	LasR	ExsA	RhlR	LecB	PqsR	PqsA
KNN 10 FT	ExsA	ExsA	ExsA	ExsA	LecB	ExsA	ExsA	ExsA	ExsA	ExsA	ExsA	ExsA	ExsA	ExsA
KNN 4 FT	PqsD	ExsA	ExsA	ExsA	LecB	ExsA	ExsA	ExsA	ExsA	ExsA	ExsA	LasR	ExsA	ExsA
KNN 5 FT	ExsA	ExsA	ExsA	ExsA	LecB	ExsA	ExsA	ExsA	ExsA	ExsA	ExsA	LasR	ExsA	ExsA
NN 10 FT	PqsD	LasR	ExsA	RhlR	LecB	LasR	LasR	PqsD	LasR	ExsA	RhlR	LecB	LasR	PqsA
NN 4 FT	PqsD	LasR	ExsA	LasR	LecB	PqsR	LasR	PqsD	LasR	ExsA	LasR	LasR	PqsR	PqsD
NN 5 FT	PqsD	LasR	ExsA	RhlR	LecB	PqsR	LasR	PqsD	LasR	ExsA	RhlR	LecB	PqsR	PqsA
SVM Linear 10 FT	PqsD	LasR	ExsA	PqsD	LecB	PqsD	LasR	PqsD	LasR	ExsA	LasR	LecB	PqsD	PqsA
SVM Linear 4 FT	PqsD	LasR	ExsA	PqsD	LecB	LasR	LasR	PqsD	PqsA	ExsA	LasR	LecB	LasR	PqsA
SVM Linear 5 FT	PqsD	LasR	ExsA	PqsD	LecB	LasR	LasR	PqsD	LasR	ExsA	LasR	LecB	LasR	PqsA
SVM Polynomial 10 FT	LasR	LasR	ExsA	PqsD	LasR	PqsD	LasR	LasR	LasR	ExsA	PqsD	LasR	PqsD	PqsA
SVM Polynomial 4 FT	PqsD	LasR	ExsA	PqsD	LecB	LasR	LasR	PqsD	PqsA	ExsA	RhlR	LecB	LasR	PqsA
SVM Polynomial 5 FT	LasR	LasR	ExsA	LasR	LasR	LasR	LasR	LasR	LasR	ExsA	LasR	LasR	LasR	PqsA
SVM RBF 10 FT	PqsD	LasR	ExsA	PqsD	LecB	PqsD	LasR	PqsD	LasR	ExsA	PqsD	LecB	PqsD	PqsA
SVM RBF 4 FT	PqsD	LasR	PqsD	PqsD	LecB	PqsD	PqsD	PqsD	LasR	PqsD	LasR	LecB	PqsD	PqsA
SVM RBF 5 FT	PqsD	LasR	PqsD	PqsD	LecB	PqsD	PqsD	PqsD	PqsA	PqsD	PqsD	LecB	PqsD	PqsA
SVM Sigmoidal 10 FT	PqsD	LasR	LasR	PqsD	LecB	PqsD	PqsD	PqsD	LasR	LasR	PqsD	LasR	PqsD	LasR
SVM Sigmoidal 4 FT	PqsD	LasR	LasR	LasR	LasR	ExsA	LasR	ExsA	LecB	LasR	ExsA	LasR	ExsA	LecB
SVM Sigmoidal 5 FT	PqsD	LasR	ExsA	LasR	LasR	PqsD	LasR	PqsD	LasR	ExsA	LasR	PqsA	PqsD	LecB
NB 10 FT	PqsD	LasR	ExsA	PqsD	LecB	PqsR	LasR	PqsD	LasR	ExsA	RhlR	LecB	PqsR	PqsA
NB 4 FT	PqsD	LasR	ExsA	LasR	LecB	PqsR	LasR	PqsD	LasR	ExsA	PqsD	LecB	PqsR	PqsA
NB 5 FT	PqsD	LasR	ExsA	LasR	LecB	PqsR	LasR	PqsD	LasR	ExsA	LasR	LecB	PqsR	PqsA
RF 10 FT	PqsD	LasR	ExsA	RhlR	LecB	PqsR	LasR	PqsD	LasR	ExsA	RhlR	LecB	PqsR	PqsA
RF 4 FT	PqsD	LasR	ExsA	RhlR	LecB	PqsR	LasR	PqsD	LasR	ExsA	RhlR	LecB	PqsR	PqsA
RF 5 FT	PqsD	LasR	ExsA	RhlR	LecB	PqsR	RhlR	PqsD	LasR	ExsA	RhlR	LecB	PqsR	PqsA
XG-Boost 10 FT	PqsD	LasR	ExsA	RhlR	LecB	PqsR	LasR	PqsD	LasR	ExsA	RhlR	LecB	PqsR	PqsA
XG-Boost 4 FT	PqsD	LasR	ExsA	RhlR	LecB	PqsR	LasR	PqsD	LasR	ExsA	RhlR	LecB	PqsR	PqsA
XG-Boost 5 FT	PqsD	LasR	ExsA	RhlR	LecB	PqsR	LasR	PqsD	LasR	ExsA	RhlR	LecB	PqsR	PqsA

As expected, predictions are more accurate for the targets with a higher number of ligands in the training database—LasR and PqsD. Furthermore, in situations when the models fail to identify the correct target, the predicted target is more represented in the dataset. Contrary to the expectation from the previous results, KNN favoured ExsA and failed to correctly pair most of the ligands and targets. The models that performed better failed to identify only PqsA, which can be explained by the high similarity of inhibitors between receptors due to their promiscuity [73].

Although the targets are very similar both in structure and function, the XGBoost and RF algorithms managed to correctly classify the ligand-target pairs. The misclassification occurred in some cases where the dataset does not have sufficient information to validate the ligand accurately or the ligand used shows promiscuity to other targets. The developed pipeline is now ready to be applied as more data become available and more inhibitors are characterized.

The accuracy of our predicted results was in line with a previous comparison of ML methods for different types of datasets, where both the selected descriptors and algorithm implementation were crucial to obtain high values of the cross validation metrics [71, 72]. The cross-validation allowed almost perfect separation for the targets represented in the database considering all the classes (Table 4).

**Fig. 5:**
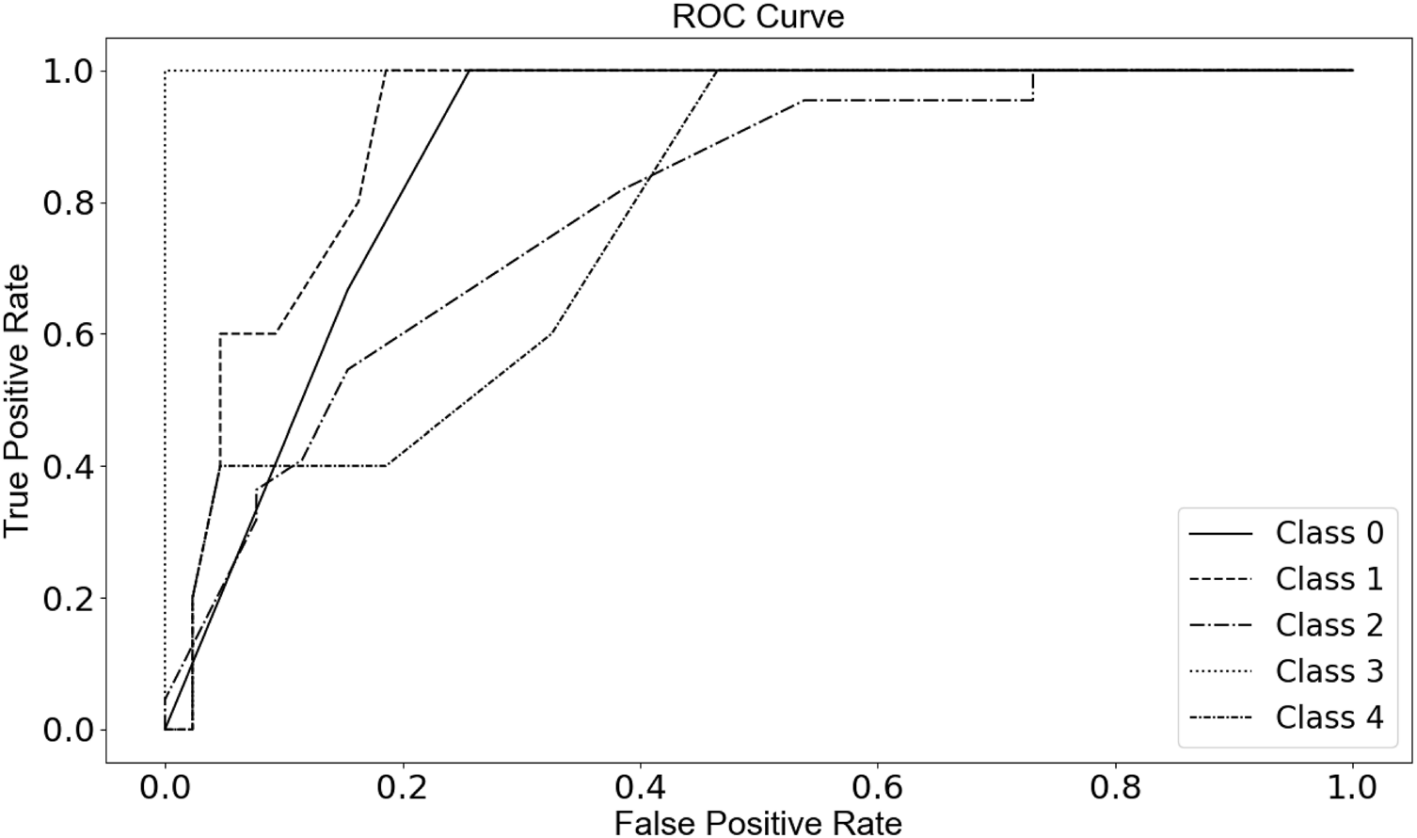
ROC One-vs-rest curve for the *5 target (gene) classes with higher number of samples* considering the best performing model (gradient boosting) for the training SigMol dataset. The graph shows the mean of the true positive rate (TP rate) and the false positive rate (FP rate)

We also tested our workflow using the SigMol database as a positive control using four features. We obtained results that showed lower AUC values for the training dataset (< 0.91) and for the testing dataset (< 0.92) for all tested models. We used the features that revealed more information gain for all models tested. The models that performed better under these conditions were the random forest (AUC = 0.91), XGBoost (AUC = 0.90), AdaBoost (AUC = 0.88), and neural network (AUC = 0.875) models. The cross-validation returned the Neural Network model as the best performing model with an AUC value of 0.85. Using the database that represents the quorum sensing molecules for the vast majority of prokaryotic organisms (1372 molecules) with a number of categories obtained for the protein targets tenfold higher (*n* = 103 recipient genes) than the number of targets in the *Pseudomonas aeruginosa* database (*n* = 7), we obtained lower AUC values. Since the optimization of the algorithm for our initial database used only four features, we tested the prokaryotic organisms database for 10 optimized features. This procedure revealed no significant variation in the AUC values, showing values for all models between 0.72 and 0.91 for the training dataset and between 0.74 and 0.92 for the test dataset. The cross-validation model that performed better was the neural network (AUC = 0.86). Nevertheless, some of the protein targets (e.g., *lasR*) included in our *P. aeruginosa* database showed an ROC curve in the prokaryotic dataset with a higher true positive rate and lower false positive rate (Fig. 5).

### Algorithm applicability for large scale datasets

The goal of the developed classification models is their application in situations where the specific protein target for an experimentally confirmed inhibitor is unknown. In experimental settings, the ability to determine the molecular protein target would enable further directed studies to design new derivatives of the initial molecule, optimizing structures to improve its affinity to the target.

To test the large-scale applicability of the workflow, several *P. aeruginosa* biofilm-inhibiting compounds were downloaded from the aBiofilm database. After descriptor calculation, these were submitted to the better performing classification models that attributed a target to each compound, as represented in Table 6. The compounds are identified by their ID code from the database.

**Table 6:** Prediction results for several *Pseudomonas aeruginosa* biofilm-inhibiting compounds in the aBiofilm database submitted to the better performing classification models

Compound	NN 10 Feat	NN 5 F	NB 10 Feat	RF 10 Feat	RF 4 Feat	RF 5 Feat	XG-Boost 10 F	XG-Boost 4 Feat	XG-Boost 5 Feat	Most Likely Targets
Anti-Biofilm_0001	LasR	LasR	LasR	LasR	LasR	LasR	LasR	LasR	LasR	LasR (100%)
Anti-Biofilm_0002	LasR	LasR	LasR	LasR	LasR	LasR	LasR	LasR	LasR	LasR (100%)
Anti-Biofilm_0003	RhlR	LasR	LasR	LasR	LasR	LasR	LasR	LasR	LasR	LasR (89%), RhiR (11%)
Anti-Biofilm_0006	PqsD	PqsD	PqsD	PqsD	PqsD	PqsD	PqsD	PqsD	PqsD	PqsD (100%)
Anti-Biofilm_0007	PqsD	PqsD	PqsD	LasR	LecB	PqsD	PqsD	LecB	PqsD	PqsD (67%), LecB (22%), LasR (11%)
Anti-Biofilm_0008	LecB	LecB	LecB	LecB	LecB	LecB	LecB	LasR	LasR	LecB (78%), LasR (22%)
Anti-Biofilm_0009	PqsD	LasR	PqsD	PqsD	PqsD	RhlR	RhlR	LasR	PqsD	PqsD (56%), LasR (22%), RhIR (22%)
Anti-Biofilm_0010	LecB	LecB	LecB	LecB	LecB	LecB	LecB	LecB	LecB	LecB (100%)
Anti-Biofilm_0011	PqsD	PqsD	PqsD	PqsD	PqsD	PqsD	PqsD	PqsD	PqsD	PqsD (100%)
Anti-Biofilm_0012	PqsD	PqsD	PqsD	ExsA	PqsD	LasR	PqsD	PqsD	PqsD	PqsD (78%), ExsA (11%), LasR (11%)
Anti-Biofilm_0013	RhlR	RhlR	PqsD	PqsD	PqsD	PqsD	PqsD	PqsD	PqsA	PqsD (67%), RhIR (22%), PqsA (11%)
Anti-Biofilm_0014	PqsD	PqsD	PqsD	PqsD	PqsD	PqsD	PqsD	LecB	PqsD	PqsD (89%), LecB (11%)
Anti-Biofilm_0101	PqsD	LasR	LasR	LasR	LasR	PqsD	LasR	LasR	PqsD	LasR (67%), PqsD (33%)
Anti-Biofilm_0108	PqsD	LasR	PqsD	LecB	PqsD	PqsD	LecB	LasR	LecB	PqsD (44%), LecB (33%), LasR (22%)
Anti-Biofilm_0109	LasR	LasR	RhlR	LasR	LecB	LasR	LasR	LasR	LasR	LasR (78%), LecB (11%), RhIR (11%)
Anti-Biofilm_0112	RhlR	LasR	LasR	LasR	PqsD	LasR	LasR	LasR	LasR	LasR (78%), PqsD (11%), RhIR (11%)
Anti-Biofilm_0113	LasR	LasR	LasR	LasR	PqsD	LasR	LasR	LasR	LasR	LasR (89%), PqsD (11%)
Anti-Biofilm_0354	LasR	LasR	LasR	LasR	PqsD	LasR	LasR	LasR	LasR	LasR (89%), PqsD (11%)
Anti-Biofilm_0405	LasR	LasR	LasR	LasR	LasR	PqsD	LasR	PqsD	PqsD	LasR (67%), PqsD (33%)
Anti-Biofilm_0406	LasR	LasR	LasR	LasR	LasR	LasR	LasR	LasR	LasR	LasR (100%)
Anti-Biofilm_0579	PqsD	PqsR	LasR	LasR	LasR	LasR	LasR	LasR	LasR	LasR (78%), PqsD (11%), PqsR (11%)
Anti-Biofilm_0588	ExsA	PqsA	LasR	LasR	LasR	LasR	LasR	PqsD	LasR	LasR (67%), ExsA (11%), PqsA (11%), PqsD (11%)
Anti-Biofilm_0755	LasR	LasR	LasR	LasR	PqsD	LasR	LasR	LasR	LasR	LasR (89%), PqsD (11%)
Anti-Biofilm_0712	LecB	LecB	LecB	LecB	LasR	LecB	LecB	RhlR	RhlR	LecB (67%), RhIR (22%), LasR (11%)
Anti-Biofilm_1133	PqsD	PqsD	PqsD	PqsD	PqsD	PqsD	PqsD	LecB	PqsD	PqsD (89%), LecB (11%)
Anti-Biofilm_1156	PqsD	LasR	PqsD	PqsD	PqsD	PqsA	PqsD	PqsD	PqsD	PqsD (78%), PqsA (11%), LasR (11%)

This screening shows that in most cases, the nine models selected are in high agreement, indicating a considerable probability of protein‒target prediction for each ligand. Furthermore, as the available data increase, the potential of this methodology to correctly identify the other targets could significantly increase, as the new results will further enable the improvement of the ML models used. Our computer-aided drug design (CADD) analysis based on ML models can be used as a complement to the omics approaches used to understand biofilm biology, such as metagenomics, transcriptomics, metabolomics, and proteomics [74]. Our model is even more relevant, as most human clinical and therapeutic inhibitors have general/broad-spectrum applications (e.g., chlorhexidine or cefazolin), which impose a more target-directed approach for specific biofilm-forming species and therapeutic applications. Some applications of these target-specific inhibitors are already in clinical trials [75, 76], showing that using the right models could expand this type of approach to biofilms.

### Example of Use

**Fig. 6:**
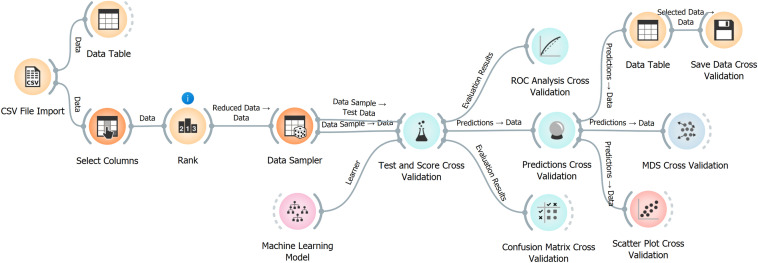
Workflow of machine learning models implementation in Orange software

The implementation of the machine learning models using an Orange software workflow was used to simplify the evaluation of the different models (Fig. 6). To replicate this methodology using our database, the following steps can be followed:

1. The database file of *P. aeruginosa* molecules with antibiofilm activity should be downloaded from https://github.com/BioSIM-Research-Group/TargIDe.
2. Download the automatic workflow implementation file (Orange software *.ows file) from the same location.
3. Load the workflow file in Orange software.
4. Import the database file using the “import csv file” widget.
5. The calculations will run automatically.
6. The results can be visualized using ROC analysis and a confusion matrix.

## Conclusions

Biofilms are an emergent issue that contributes to bacterial multidrug resistance. The urge to develop new and target-based drugs has led to a shift in the drug-design paradigm in the last decade, further inspiring collaborations between experimental and theoretical studies. Frequently, promising anti-biofilm inhibitors for different bacteria are identified, with no knowledge of the precise protein target on which they are acting. A correct identification of the protein target directly involved in the inhibitory activity of a molecule is essential for optimization of its activity through the development of new derivatives with improved target affinity. In this work, we propose a workflow to correctly identify the most likely protein targets of molecules with confirmed *P. aeruginosa* anti-biofilm activity.

Combining CAAD techniques such as database curation, chemical descriptor calculation, feature selection, machine learning classification model development, and database screening, the optimized workflow is now ready to be applied to new molecules, as more data become available and characterized. The developed workflow can easily be adapted and applied to other biological and chemical issues, suggesting a new way of approaching the initial issues of antibiofilm drug design.

## Electronic supplementary material

Below is the link to the electronic supplementary material.

Supplementary Material 1

## Data Availability

The implemented algorithm and data are freely available at https://github.com/BioSIM-Research-Group/TargIDe under licence GNU General Public Licence (GPL) version 3.
